# Circular Economy Insights on the Suitability of New Tri-Layer Compostable Packaging Films after Degradation in Storage Conditions

**DOI:** 10.3390/polym15204154

**Published:** 2023-10-19

**Authors:** Ricardo Ballestar de las Heras, Sergio Fernández Ayala, Estefanía Molina Salazar, Fernando Carrillo, Javier Cañavate, Xavier Colom

**Affiliations:** 1 Research Department of Sphere Group Spain, P.I El Pradillo 3 C/Sphere, Parcela 9, 50690 Pedrola, Zaragoza, Spain; rballestar@sphere-spain.es (R.B.d.l.H.); sfernandez@sphere-spain.es (S.F.A.); mprima@sphere-spain.es (E.M.S.); 2 Department of Chemical Engineering ESEIAAT, Universitat Politècnica de Catalunya BarcelonaTech. Colom 1, 08222 Terrassa, Barcelona, Spain; fernando.carrillo@upc.edu (F.C.); francisco.javier.canavate@upc.edu (J.C.)

**Keywords:** circular economy, biodegradable compound, packaging, tri-layer film, PBAT, FTIR, DSC, colorimetry

## Abstract

The environmental degradation of the films used in packaging is a key factor in their commercial use. Industrial and academic research is aimed at obtaining materials that have degradation features that ensure their eco-sustainability but, at the same time, preserve their use properties during storage and distribution periods. This study analyzes the degradability behavior over time of commercial packaging that meets the requirements of the UNE 13432 standard and the prEN 17427 (2020) home composting certification requirements under standard storage conditions. The study attempts to provide insight into the durability of the films under standard storage conditions, verifying that this type of packaging has a useful life of more than 12 months and that after this storage period it still retains the usability properties for which it was conceived. The analyzed sample has been manufactured using a three-layer technology under some commercial formulations based on PBAT + STARCH + PLA and has been analyzed monthly for 12 consecutive months. The macroscopic monitoring of the degradation of the sample has been carried out through the evolution of the mechanical properties and the quantification of the color changes (very important in films) via colorimetry. The nature of the observed variations has been justified at the microstructural level from the data obtained in calorimetric analysis (DSC) and from the characterization using FTIR. The results indicate a loss of properties in the tensile, elongation and impact tests and a behavior of stability or improvement in the tear properties of the film. Analyzing the microstructural changes, it is observed that the degradation of a hydrolytic and thermo-oxidative type occurs in the amorphous part of the film. The conclusion of this study is that the proposed packaging, focused on domestic composting and stored under standard conditions, has a useful life of more than 12 months. This period should be sufficient to cover the stages of production, storage and final use.

## 1. Introduction

Today, plastic consumption has increased exponentially at an estimated global annual production rate of almost 400 million tons [1]. Bio-based, biodegradable and compostable plastics currently represent 1% of the global European plastics market. An overall 5–8% growth of these more environmentally friendly plastics between 2020 and 2025 is expected in Europe, where the packaging sector makes up 40% of plastic production and is the leader in market share [2].

The current socioeconomic system is based on a linear economy, which implies the generation of waste, energy outflow and noticeable degradation of the environment However, plastic containers also have good qualities; they are lighter and more durable compared to other options [3,4,5].

**Figure 1:**
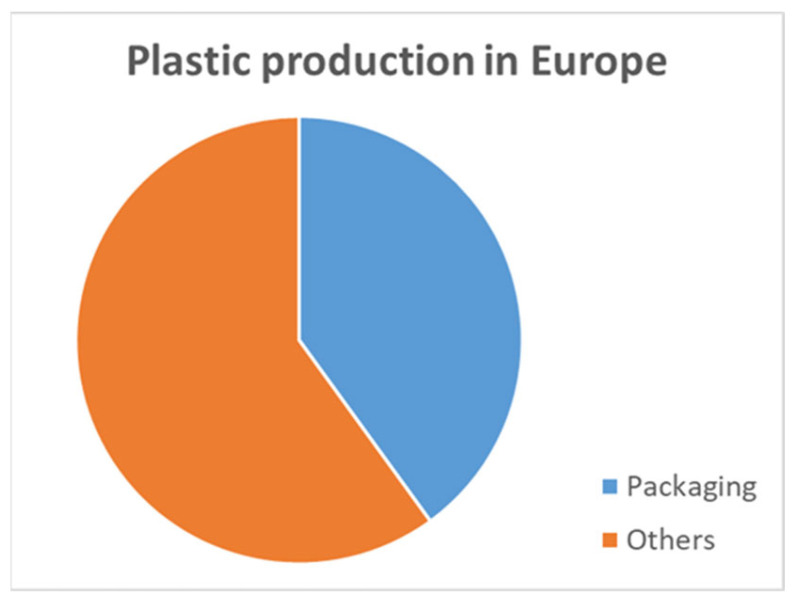
Sectorial plastic production in Europe (redrawn ref. [3]).

Since 3 July 2021, when the SUP (Single-Use Plastic) directive EU 2019/904 was implemented, a legislative movement toward compostable plastics has developed throughout Europe, aligned with the “Green Deal” of 2020 [6,7], with the final goal of climate neutrality by 2050. In Spain, this European directive was transposed on 8 April 2022 with Law 7/2022 [8,9] on waste and contaminated soils for a circular economy. Article 28 of this regulation is aimed at clarifying the different types of plastics related to packaging and their classification in order to proceed with selective recycling (Figure 1).

The trend toward packaging plastics that are in consonance with the concepts of circular economy is encouraged, not only by European legislation but also by different international policies around the world, including China, the USA, India, Japan, Malaysia, Indonesia, Canada and others [10].

To align the current packaging films with the new regulations, there is a necessity for the development of novel films that meet the criteria of being biodegradable, compostable, and in full compliance with all legal mandates [11].

The term biodegradable implies that the material can be decomposed by microorganisms, but not necessarily that it is compostable [12]. A compostable material is considered when the compost it produces is of good quality and is safe for use as a fertilizer for plants without damaging the cultivated soil [13].

Evidently, biodegradability and compostability depend, to a great extent, on the conditions and environment in which the material is degraded. This shares similarities with other plastic materials [14]. Variables such as the type of microorganisms existing in the soil, temperature, moisture and others can substantially affect the degradation process. Plastics that are biodegradable in a composting industrial plant, where the conditions are aggressive, cannot be biodegradable in regular conditions, as when deposed in a composting container in a garden [15].

The use of films that present features such as biodegradability can enormously reduce their environmental impact. The use of such films for applications with high consumption in agriculture could help to decrease the presence of microplastics in soil. It is estimated that biodegradable and compostable plastics could absorb approximately 50% of global production [16].

However, being biodegradable a desired feature in these plastics, can in many cases also mean a decrease in storage time, an uncontemplated decrease in mechanical properties or unexpected changes in visual appearance. For this reason, an assessment of the degradation processes that take place in such materials is very important. At the same time, a better knowledge of the changes in the properties of the material would be achieved when the microstructural processes, degradation reactions and their relation to the macroscopic effects were properly researched [17].

**Figure 2:**
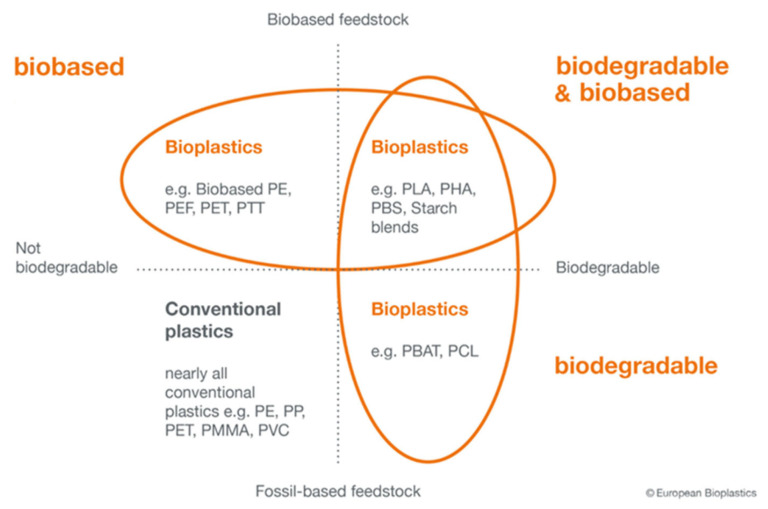
Classification of plastic materials according to their origin and biodegradability. Redrawn from image source: European Bioplastics e.V. (EUBP).

Previous works have already adopted this approach, studying films based on starch [18]. Starch, because of its low price, biodegradability and availability, is considered a good substitute for synthetic plastics for some applications. Interesting data on the biodegradation of aliphatic–aromatic copolyesters and their ecotoxicology have also been published [19]. Derivatives of polylactic acid are also considered for packaging [20]. In the context of food packaging, a methodology for scoring and comparing environmental effects has also been developed [21]. A general classification of some plastics according to their biodegradability and composition is presented in Figure 2.

In this work, a commercial packaging film of known composition, obtained by finely tuned extrusion combining technology and materials, accredited according to the previously cited regulations, is chosen as the subject of study.

The formulation is based on a blend of polybutylene adipate terephthalate (PBAT), commercial starch compound, commercial PLA compound and mineral charge.

These polymers are compostable and have been drastically increasing in consumption in recent years. Companies such as BASF, Biotec and Novamont have developed several products related to these materials.

To observe Directive 94/62/CE, the films must pass tests and meet the following criteria: (i) no heavy metals and less than 50% volatile solids; (ii) 90% biodegradability; (iii) fragments of degraded material must be smaller than 2 × 2 mm within 2 weeks and (iv) the resulting compost must be non-ecotoxic.

PBAT has been used in the production of blown films, and similar polymers have been studied in format blown films, blends with montmorillonite, blends polylactide (PLA)/PBAT and PLA blends [22,23,24,25]. The PLA/poly(butylene adipate-co-terephthalate) blends have also been studied from the point of view of biodegradation [26,27,28].

Nevertheless, not many studies on the biodegradability of real commercial blends are available in the literature, and most of them are related to the research of the degradation of the materials from the point of view of their effects on the environment. There is a lack of knowledge on the effects of the biodegradability of the new polymeric films regarding their usability. If the biodegradable films are called to replace traditional synthetic films, the following questions need to be answered: can the biodegradable polymers be stored before use without compromising their final properties? How can the storage conditions and composition affect the degradation? Will the bags stored typically for months still be in good condition to be used by consumers? 

In this manuscript, the commercial films based on a commercial starch compound and PLA compound + mineral charge cited above have been subjected to degradation in real storage conditions. The changes in the samples have been monitored by the determination of their mechanical properties, (according to ISO 527 [29], ISO 7765-1 [30], ISO 6383-2 [31]), by thermal analysis, (DSC) and by FTIR. In films, degradation may appear in the form of discoloration or yellowness. For this reason, a colorimetric study of the samples, initially transparent and colorless, has also been performed.

The primary objectives were twofold: firstly, to assess the packaging’s suitability under storage conditions one year after production while establishing an optimized “best before” date for these products in the market. Secondly, to comprehend the microstructural alterations occurring in the samples that subsequently influence macroscopic properties through the application of FTIR and thermal analysis. This approach enhanced our understanding of the material and paved the way for tailoring films to meet specific application requirements in the future.

## 2. Materials and Methods

### 2.1. Film Composition

**Figure 3:**
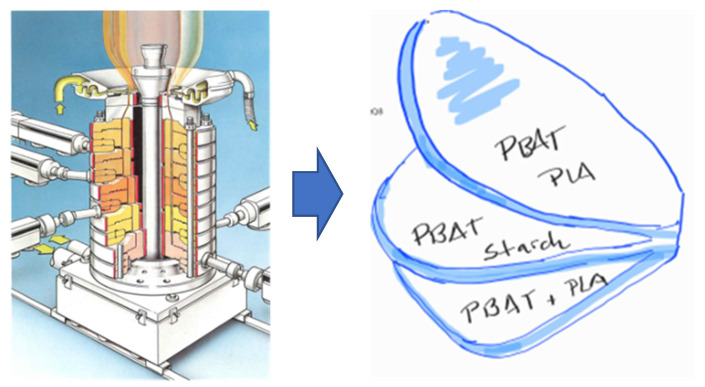
Manufacturing process and graphic scheme of tri-layer film (Source: HOSOKAWA MANUAL).

In order to achieve the complex properties necessary for these films, a combination of selected materials and a special technology have been applied. The films are made using a tri-layer assembly, where the external layers are composed of a biocompostable fossil-based material and the internal layer is composed of commercial starch and PLA compounds (Figure 3). This type of structure, obtained via coextrusion, combines the desired biodegradability with the mechanical properties required for the final use.

The formulation of the final product is shown in Table 1.

The material in the central layer is, as mentioned above, a commercial blend starch compound. It is a plasticizer-free and GMO-free thermoplastic material that is based on natural starch and suitable for processing via blown-film extrusion to produce items that are completely biodegradable and compostable according to EN 13432 [32]. It has been supplied by a European company. Starch is a combination of amylose and amylopectin, two very similar polysaccharides [33].

In the starch, the content of amylopectin comprises between 70% and 80% by weight, independent of the size of the granules.

This starch compound has a density of 1.26 g/cm^3^ and an MFI (190 °C/5 kg) of 8.40 g/10 min. The PLA compound used is also provided by the same supplier; it has a density of 1.24 g/cm^3^ and MFI (190 °C/2.16 kg) of 4.10 g/10 min. The PBAT has a density of 1.22 g/cm^3^ and MFI (190 °C/2.16 kg) of 3.70 g/10 min. The multilayer film was produced with a coextruder Reifenhäuser Blown Film Polyrema (Reifenhäuser Blown Film GmbH & Co., Troisdorf, Germany) with extruders type 60F/70F/60F–30D V3 blow-up ratio 3.90 and average thickness of 14 microns.

### 2.2. Film Degradation

The film has been stored under standard conditions in Zaragoza, Spain (latitude: 41°39′38″ N–longitude: 1°0′15″ O) from 1 February 2021 (reference sample) to 31 January 2022. The meteorological parameters are presented in Table 2 and have been supplied by AEMET (Spanish Agency of Meteorology).

### 2.3. Film Characterization

The mechanical properties of the samples were measured monthly over the course of 12 months. The tests performed were impact, tensile strength and tearing.

The impact properties were evaluated using a dart test according to ISO 7765-1 with Metrotec equipment (Metrotec, Lezo, Spain) in order to characterize the fracture behavior against impact dart loads. Enough distance was kept between successive tests in order to ensure no influence from previous impacts. The total mass was adjusted with the staircase method that applies to the mentioned standard, whose minimum objective is 10 breaks. The conditions were as follows: impact radius 8 cm, dart weight 25 g/50 g/100 g, added weights 5 g/15 g/30 g/45 g/60 g/90 g/100 g, minimal number of breaks, as said above, was 10.

Tensile strength and elongation at break of the samples were tested according to the standard ISO 527, considering the orientation of the test tube, using an IDM testing machine (0301N208) IDM Test (Ingeniería y Desarrollo de Máquinas, S.L. San Sebastian, Spain) with a cell load capacity of 250 N. Tensile tests were performed at a cross-head speed of 500 mm/min. Direct extension measurements were conducted periodically using an extensometer with sensor arms. 16 replicates of the test are carried out in each direction analyzed, with a constant width of the specimen of 15 mm and a distance of 50 mm between the grips.

The Elmendorf tear properties of all blown films were measured using an IDM Elmendorf DEA-80 Tear tester IDM Test (Ingeniería y Desarrollo de Máquinas, S.L. San Sebastian, Spain) following the standard ISO 6383-2. Two film sections of 76 mm × 63 mm were cut with a sample cutter from each test film produced and their thickness measured. A 20 mm slit was made at the center of the edge perpendicular to the direction being tested. Eight replicates of each test were performed in each direction.

A colorimetric study has also been performed on the samples, which were originally transparent and colorless. The measurements were made with a PCE-CSM-2 colorimeter (PCE Instruments, Meschede, Germany), determining the tristimulus values of 16 stacked layers of samples on a white surface. In order to evaluate the differences, the L*a*b* CIELAB color space is taken as a reference. The three coordinates of CIELAB represent the lightness (L* = 0 black and L* = 100 white), the balance red/green (a* green = negative; red = positive) and position yellow/blue (b*, blue = negative; yellow = positive). The asterisks (*) after L*, a*, and b* are pronounced stars and are part of the full name to distinguish L*a*b* from Hunter’s Lab. [34]

The calorimetric analysis was performed using a Mettler DSC1 calorimeter (Columbus, OH, USA) calibrated using indium (heat flow calibration and temperature calibration) and zinc (temperature calibration) standards. Samples of approximately 10 mg of the mass were deposited in 40 µL aluminum pans in an air atmosphere to test their performance. The sample is heated at a rate of 10 °C/min to 180 °C to erase the thermal history of the processing. After that, it is cooled to 30 °C to determine crystallinity and left for 5 min for stabilization; after that, it is heated again to 180 °C to determine melting temperature.

The chemical structure of the compound-degraded samples was determined using FTIR analysis performed by means of a Spectrum Two spectrometer from Perkin Elmer (Waltham, MA, USA). The device had an ATR attachment with a diamond crystal.

Spectra were registered at a 2 cm^−1^ resolution and 40 scans in the range of 500–3500 cm^−1^, in which the compound signals related to different deformation bands can be observed.

To ensure the reproducibility of our results, we conducted multiple measurements for each test. We performed impact testing until at least 10 breaks occurred. Tensile strength and elongation at break were assessed using 16 specimens in each direction. Tearing tests were replicated eight times for each sample. Colorimetric measurements were obtained by measuring 15 stacked film layers. Calorimetric analysis was performed following standard procedures, with one sample of each type. Spectra were obtained via ATR, averaging 40 scans per spectrum. In the figures presented, the standard deviation is represented by error bars associated with each data point.

## 3. Results

### 3.1. Technical Requirements of Films

This type of packaging film is subject to technical requirements determined by the market that have been implemented in the company according to the technical specifications in Table 3. These values are concerted in relation to quality assurance and specify the minimum value required for each of the tests in order to not compromise the usability of the final product according to the company standards. The table presents the technical specifications required for a 14-micron film made of biodegradable and compostable materials, in accordance with the EN 13432 standard, for use in the packaging of food and vegetable (F&V) end products.

### 3.2. Mechanical properties

Table 4 shows a short summary (exact data) of the results of the mechanical tests along the studied period (12 months).

#### 3.2.1. Impact Resistance

The results of the impact resistance can be seen in Table 4. There is a decrease in the impact resistance of around 30% during the 12 months that the thermo-oxidative degradation lasts. Comparing the results with the requirements of the compostable material (Table 3), the impact value should still be considered acceptable at the end of the test. However, the rather drastic reduction to a value of 14 g/µm (compared to the minimum requirement of 10 g/µm) may raise concerns about the suitability of a compostable material that is very sensitive to hostile environments, stored for longer periods or under more aggressive conditions.

#### 3.2.2. Tensile Strength

**Figure 4:**
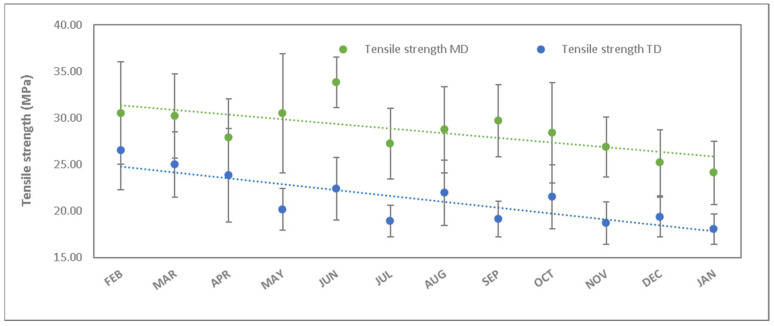
Tensile strength as a function of the studied period (green values corresponds to longitudinal direction and blue values to transverse direction).

The tensile strength values presented in Table 4 and Figure 4 show a decrease of 24% in the longitudinal direction and 34% in the transverse direction for the studied degradation period. As can be seen, the two types of degradation processes (longitudinal direction and transverse direction) are very similar. This is because the thermo-oxidative process with a relative humidity (RH) of 55% affects the structure in a similar way. The reactions that take place are of type Norrish I and II [35] where there are several thermos-oxidative processes, including the formation of carbonyl groups, the cleavage of hydrocarbon chains, the formation of free radicals in tertiary carbons, and the formation of unsaturated bonds followed by branching and possible crosslinking [36,37].

**Figure 5:**
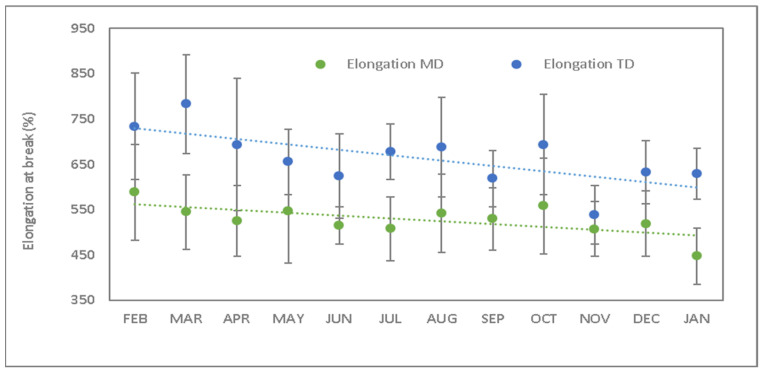
Elongation at break during the studied period (green values corresponds to longitudinal direction and blue values to transverse direction).

Figure 5 shows the elongation at break values in both directions, giving a diminution of 24% longitudinal and 14% transverse. According to reference values (Table 3), the elongation minimum value was set at 250% and transverse elongation at 400%. The obtained values after degradation are 447% and 629%, which means that the decrease in elongation should not be a concern for the final use.

Unlike tensile strength (TS), elongation at break (EB) defines different behaviors depending on the direction of the test. The longitudinal direction gives lower EB values than the transverse direction. However, thermo-oxidative degradation in the longitudinal direction is lower than the transverse one. This is because the manufacturing process of this type of film causes stretching in the longitudinal direction, aligning the macromolecules. This fact increases the difficulty of breaking the macromolecules themselves as well as the reduction in the deformation of the material’s own structure. At the same time, the compacity achieved in this direction may make the penetration and the exposition to the environment of the internal layers difficult. According to Table 1, tri-layer films are mostly made up of PBAT (with a soft/aliphatic area in the compound), which implies that the degradation process takes place mainly on this component. It is responsible for the structural changes that take place in this material.

#### 3.2.3. Tear Resistance

**Figure 6:**
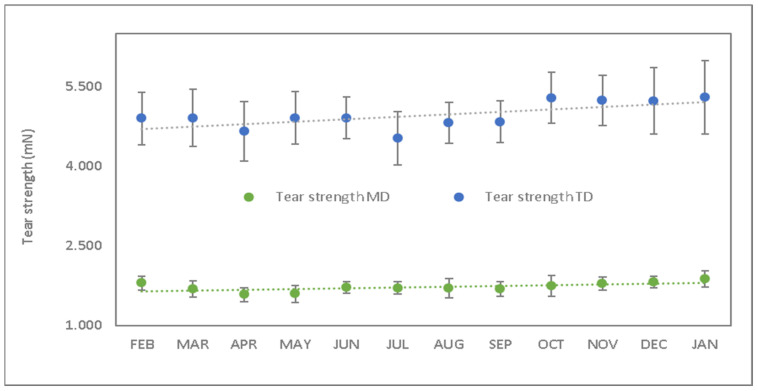
Tear strength as a function of studied period (month) (green values corresponds to longitudinal direction and blue values to transverse direction).

Another interesting mechanical property for this type of material is the tear strength. Contrarily to the previous results, it shows a positive slope of the graph (Figure 6) with an increase in the tearing of about 4% longitudinally and 8% transverse.

The Longitudinal Tear Strength (LTS) observed in Figure 6 shows a different behavior depending on the period of time of exposure to the degradation process. During the first 8 months, stable behavior appears with a decreasing trend. In contrast, during the last 4 months there has been a significant increase (from 4.9 N to 5.3 N), which remains stable.

The tear resistance test is determined with a system in which the tear resistance of the structure of the film of compostable material is measured. This resistance is the force perpendicular to the material necessary to propagate the tear until it cuts the specimen by applying an instantaneous force (milliseconds). The fact that the applied force is instantaneous determines the structural behavior of the film. As previously mentioned, the thermo-oxidative degradation process generates the formation of unsaturated bonds, followed by branching and possible crosslinking. The crosslinking that takes place during this degradation stiffens the material, and consequently, the work required to tear the sample increases as a function of the exposure period. In this case, during the first months, no crosslinking has occurred yet, and consequently, an increase in tear resistance is not perceived. It is from the eighth month on that this phenomenon is significantly observed.

### 3.3. Structural Characterization with FTIR

**Figure 7:**
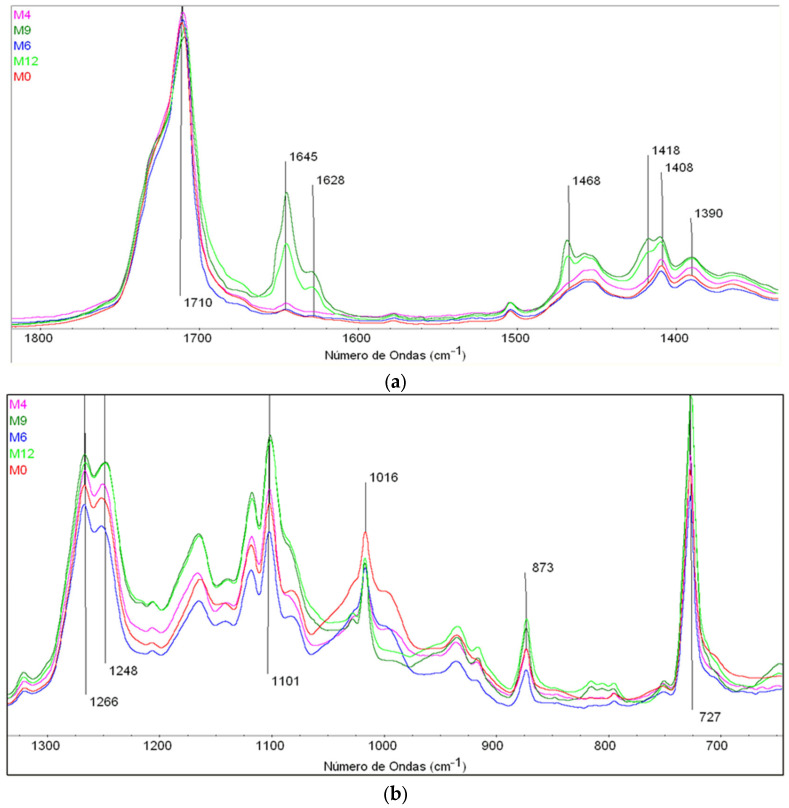
(**a**) Spectral area between 1800 and 1300 cm^−1^. (**b**) Spectral area between 1300 and 600 cm^−1^.

Figure 7a shows the spectral area between 1800 and 1300 cm^−1^. In this part of the spectrum, the bands of 1750 and 1710 are assigned to the C=O groups of the PLA and the PBAT, respectively. In both, a decrease is observed as a function of the thermo-oxidative process exposure time. As it is reflected in the proposed degradation mechanism, some C=O disappear to form new OH groups.

The bands at 1645 and 1628 cm^−1^ are also clearly observed, with high intensity values at 9 and 12 months of exposure time. These bands are attributed to C=C, stating that the thermo-oxidative degradation process generates double bonds and that later some of them disappear due to the formation of crosslinking.

The 1468 cm^−1^ band, assigned to the methyl group (CH_2_), presents an identical progression to the 1645 cm^−1^ bands since the unsaturation formation implies the formation of CH_2_ and CH groups (1455 cm^−1^ band). Interesting is the doublet 1418–1408 cm^−1^, these bands assigned to vinyl groups (1418 cm^−1^ aryl vinyl CH and 1408 cm^−1^ alkyl vinyl CH), formed during the thermo-oxidative degradation process in the adipate terephthalate linking zone. 1390 cm^−1^ assigned to CH_3_ rises due to the exposure period. These observations about the FTIR bands align with the findings of other researchers [38].

Analyzing the spectral area in Figure 7b from 1300 to 600 cm^−1^, it observes the change of bands at 1266 and 1248 cm^−1^ (assigned to C-O, and COH), while the group CO remains constant and the COH increases due to chain breakage and OH group formation. The band at 873 assigned to the calcium ion remains constant and the intense band of 727 cm^−1^ corresponds to CH_2_.

**Figure 8:**
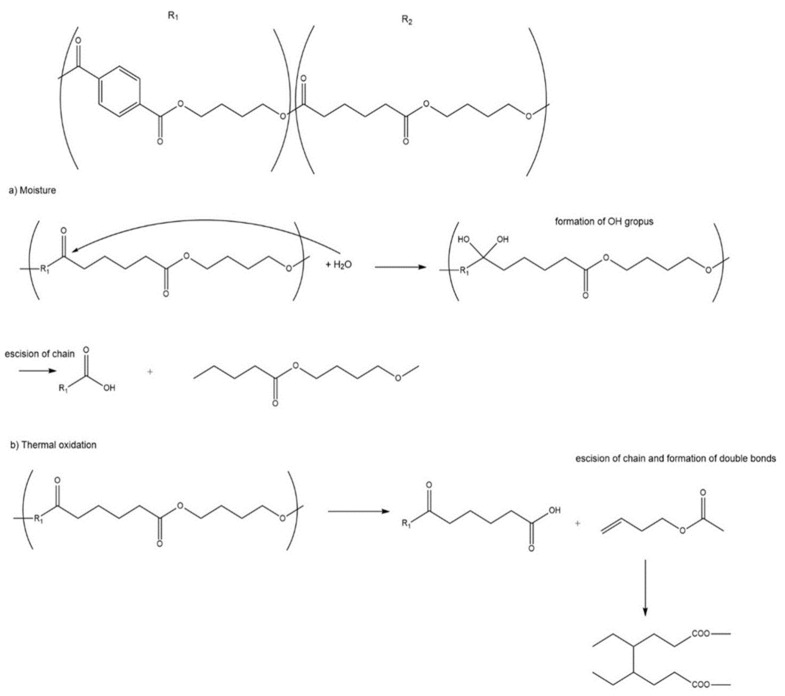
Chart of the mechanisms of the thermo-oxidative degradation process.

Thermo-oxidative degradation mechanism of PBAT is observed in Figure 8.

### 3.4. Colorimetry

Table 5 shows the colorimetric results obtained during 12 months of thermo-oxidative exposure, and Table 6 shows the difference between the initial and final values of the parameters L*, a* and b* as well as the color difference calculated with the general formula:

$${∆E}_{}^{*}=\sqrt{{\left(L_{2}^{*}-L_{1}^{*}\right)}^{2}+{\left(a_{2}^{*}-a_{1}^{*}\right)}^{2}+{\left(b_{2}^{*}-b_{1}^{*}\right)}^{2}}$$

**Figure 9:**
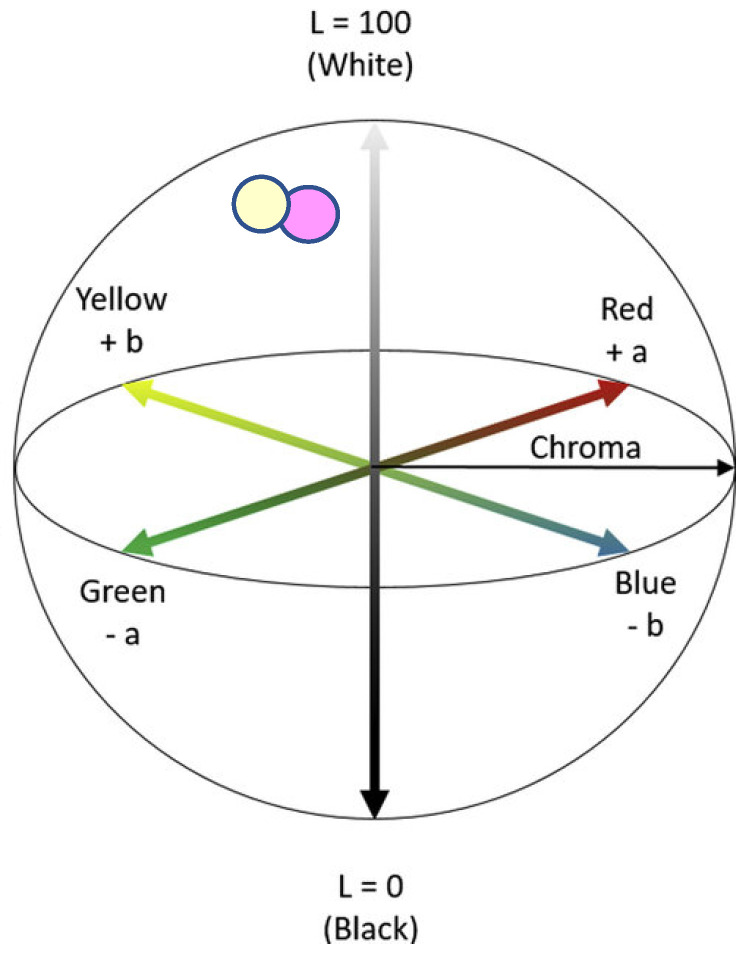
CIELAB color space diagram of initial and final lightness (L*) chromaticity parameters (a*,b*). Purple circle corresponds to initial value and yellow circle corresponds to the final value.

Practically no difference in color between both values is observed. Figure 9 shows, in the CIELAB color space diagram, the two close positions in which the initial and final values are located. The final sample tends to be whiter (less transparent), but the variation is not perceptible to the eye, even overlapping several layers of film.

### 3.5. Thermal Analysis

**Figure 10:**
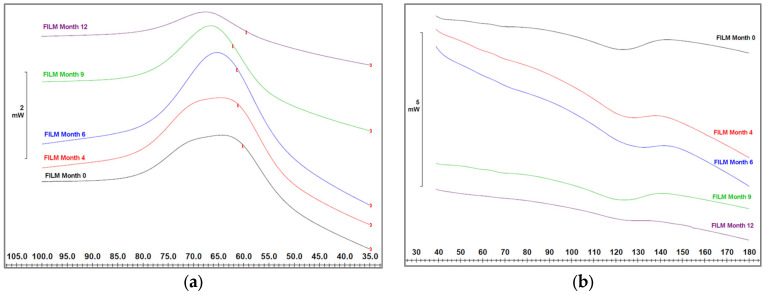
(**a**) DSC cooling (crystallization peak) and (**b**) DSC heating (melting peak) as a function of studied period (month).

Figure 10a shows the cooling, and Figure 10b shows the two on run heating thermograms obtained with DSC of the different degraded samples over 12 months. As can be seen, the crystallization and melting temperatures of the PBAT sample are 62–70 °C and 123 °C before degradation. As the samples degrade, the temperatures change in crystallization and increase in melting. The modification in crystallization temperature goes from a broad peak of 70–62 °C that appears in the first months to 68 °C. From month 6 onwards, this broad band becomes a single peak. The increase in melting temperature goes from 123 °C to 132 °C. During the first months, the shift is significant, and as the degradation process lengthens, the peak attenuates until it disappears completely.

The high content of PBAT in the sample allows the consideration that all thermal phenomena that take place are mainly due to the PBAT compound. PBAT is a biodegradable polyester with a high content of amorphous structure and low-defined melting peaks, making the amorphous structure very susceptible to hydrolytic degradation than crystalline regions. PBAT consists of butylene adipate (BA) units with higher mobility and stiffer butylene terephthalate (BT) units that share a common mixed crystalline structure surrounded by mostly amorphous structures.

Hydrolytic degradation breaks the chains of the amorphous aliphatic area and allows a reorientation that slightly increases the melting temperature. Also, when the degradation is more severe, it affects the mixed crystalline area, and this is when practically the melting peak disappears.

The modification of crystallization temperature corroborates the chain breakage of the aliphatic zone of PBAT that takes place in the degradation process. During the first months of degradation, the broad band becomes a peak with a tendency of decreasing crystallization temperature. This is due to the fact that the mobility of the short chains consolidates at lower temperatures. When the degradation is more severe, the possibility of consolidating crystalline structure will only be in the aromatic area of the PBAT, and the crystallization peak will be lower and at a higher temperature.

## 4. Conclusions

Analyzing the results of mechanical properties, it seems that the proposed biocompostable material, in regular storage conditions without any extreme weathering environments, can be altered in its mechanical properties during a period of one year. This may raise concerns about the conservation of these films pre-use and also recommends providing a caducity date for these materials.

From the point of view of color change (very important in the case of packaging), the degradation shows no visible modification of the material.

Eventually, the final properties will still fulfill the requirements. The film is useful after this test. The main issue here is that while synthetic films were stored without any other considerations about caducity, the data show that perhaps, in more aggressive environments, the biocompostable films could be altered quite significantly.

The microstructural studies performed with FTIR and DSC show that the main degradation occurs in the PBAT and, concretely, in the aliphatic chains.

As in other polymers, the main degradation phenomena involved are the breaking of chains (aliphatic parts of the PBAT), the formation of double bonds and the apparition of oxygenated products of the degradation. If the degradation period is long (or intense) enough, the formed double bonds can undergo crosslinking reactions that increase the fragility of the polymer.

As a consequence of this research, a new avenue of investigation emerges concerning the environmental conditions under which these films can be stored and the potential establishment of conditions for their preservation.

Moreover, considering the global context and taking into account current consumer preferences and their concerns [39], we will propose a new study with the aim of examining the fragmentation and degradation of the film in various scenarios: (a) the final product made from biocompostable materials exposed to outdoor conditions, (b) the final product composed of biocompostable materials submerged in water and (c) the final product constructed from biocompostable materials buried in the ground.

## Figures and Tables

**Table 1:** Formulation of the tri-layer film.

Distribution of Layers	Layer A	Layer B	Layer C
Percentage of total thickness	30%	40%	30%
PBAT	65%	10%	55%
Starch compound		75%	
PLA compound	25%	5%	35%
Mineral charge	10%	10%	10%

**Table 2:** Meteorological variables during the aging period (2021–2022).

	Feb.	Mar.	Apr.	May	Jun.	Jul.	Aug.	Sep.	Oct.	Nov.	Dec.	Jan.
**T. average (°C)**	9.4	8.4	10.8	15.4	19.8	22.7	23.1	18.8	13.3	6.1	7.0	2.0
**T. average max (°C)**	15.3	15.3	16.9	22.2	26.7	31.2	31.3	25.1	21.3	11.1	12.5	7.3
**T. average min (°C)**	3.6	1.4	4.6	8.5	12.9	14.2	14.8	12.4	5.3	1.1	1.4	−3.2
**Average rainfall (mm)**	26.0	2.4	64.8	70.8	50.0	19.0	15.6	114.4	31.8	91.2	13.4	48.0
**Relative humidity average (%)**	65	59	63	53	55	42	46	64	63	74	76	77

**Table 3:** Technical requirements of F&V biocompostable product.

Mechanical Property	Technical Specification—Minimum Value
Impact (g/µm)	10
Tensile strength LD (MPa)	9.6
Tensile strength TD (MPa)	7.89
Elongation LD (%)	250
Elongation TD (%)	400
Tearing LD (mN)	1200
Tearing TD (mN)	4000

**Table 4:** Monthly results of the mechanical tests during the studied period.

**Month**	**Feb.**	**Mar.**	**Apr.**	**May**	**Jun.**	**Jul.**
Impact (g/µm)	19.0 ± 0.52	19.0 ± 0.59	19.0 ± 0.63	18.0 ± 0.44	19.0 ± 0.47	19.0 ± 0.63
Tensile strength LD (MPa)	30.52 ± 0.41	30.24 ± 0.46	27.9 ±0.50	30.48 ± 0.58	33.83 ± 0.47	27.24 ± 0.42
Tensile strength TD (MPa)	26.53 ± 0.40	25.04 ± 0.42	23.84 ± 0.47	20.19 ± 0.50	22.4 ± 0.47	18.93 ± 0.41
Elongation LD (%)	588 ± 34.60	544 ± 41.84	525 ± 39.22	547 ± 36.78	515 ± 37.79	508 ± 39.12
Elongation TD (%)	733 ± 46.94	783 ± 49.16	693 ± 50.76	655 ± 43.66	624 ± 40.97	678 ± 49.95
Tearing LD (mN)	1799 ± 199	1695 ± 185	1582 ± 176	1595 ± 201	1718 ± 183	1707 ± 168
Tearing TD (mN)	4897 ± 242	4907 ± 216	4660 ± 267	4907 ± 230	4908 ± 236	4523 ± 233
**Month**	**Aug.**	**Sep.**	**Oct.**	**Nov.**	**Dec.**	**Jan.**
Impact (g/µm)	19.0 ± 0.52	19.0 ± 0.42	17.0 ± 0.78	17.0 ± 0.69	16.0 ± 0.67	14.0 ± 0.55
Tensile strength LD (MPa)	28.74 ± 0.43	29.71 ± 0.51	28.43 ± 0.37	26.91 ± 0.31	25.2 ± 0.30	24.12 ± 0.30
Tensile strength TD (MPa)	21.99 ± 0.40	19.17 ± 0.49	21.56 ± 0.30	18.73 ± 0.28	19.36 ± 0.32	18.07 ± 0.30
Elongation LD (%)	542 ± 32.97	530 ± 34.85	558 ± 31.11	507 ± 28.15	519 ± 32.78	447 ± 26.70
Elongation TD (%)	687 ± 45.13	618 ± 44.55	693 ± 39.16	539 ± 37.94	632 ± 41.93	629 ± 40.15
Tearing LD (mN)	1704 ± 200	1686 ± 186	1745 ± 182	1794 ± 195	1824 ± 207	1879 ± 182
Tearing TD (mN)	4816 ± 219	4836 ± 228	5288 ± 297	5234 ± 235	5227 ± 232	5297 ± 245

**Table 5:** Results of L, a and b of the samples during the studied period.

Month	Feb.	Mar.	Apr.	May	Jun.	Jul.	Aug.	Sep.	Oct.	Nov.	Dec.	Jan.
***L***	92.72	92.54	92.80	92.71	92.21	92.48	93.14	93.93	93.31	93.06	93.17	93.21
***a***	−1.92	−1.98	−1.98	−1.93	−2.01	−1.98	−2.03	−1.95	−1.97	−2.01	−2.06	−2.12
***b***	1.38	1.36	1.42	1.69	1.31	1.33	1.46	1.44	1.22	1.10	1.30	1.15
**∆*E***	0.00	0.19	0.11	0.32	0.36	0.25	0.44	1.21	0.61	0.45	0.48	0.58

**Table 6:** Summary of the variation of the colorimetric properties during the 12 months of study.

Month	Initial	Final	Variation
***L***	92.72	93.21	1%
***a***	−1.92	−2.12	10%
***b***	1.38	1.15	−17%
** ∆*E***	0	0.58	-

## Data Availability

The data presented in this study are available on request from the corresponding author.
